# IgG4-related systemic disease mimicking renal pelvic cancer: a rare case

**DOI:** 10.1186/1477-7819-12-395

**Published:** 2014-12-23

**Authors:** Yiwei Wang, Xing Chen, Rongkui Luo, Hang Wang, Guomin Wang, Yingyong Hou, Jianming Guo

**Affiliations:** Department of Urology, Zhongshan Hospital, Fudan University, 180 Fenglin Road, Shanghai, 200032 China; Department of Urology, China-Japan Friendship Hospital, No.2, Yinghua East Road, Beijing, 100029 China; Department of Pathology, Zhongshan Hospital, Fudan University, 180 Fenglin Road, Shanghai, 200032 China

**Keywords:** IgG4, Mikulicz’s disease, Renal pelvic cancer

## Abstract

**Background:**

Immunoglobulin G4–related disease (IgG4-RD) is a new clinical entity. Characteristic features of IgG4-RD are elevated serum IgG4 levels, infiltration of IgG4-positive cells, mass-forming lesions with fibrosis and good response to corticosteroids. The variable imaging features of IgG4-RD and the overlap with other differential diagnoses often pose a diagnostic challenge, as they frequently mimic malignant tumors or other inflammatory diseases in the abdomen.

**Case presentation:**

A 54-year-old woman visited our hospital with left flank discomfort and palpebral edema. Computed tomography, magnetic resonance imaging, retrograde pyelography and positron emission tomography/computed tomography indicated renal pelvic cancer. However, after a left-sided nephroureteral cystectomy was performed, the mass was pathologically confirmed as an IgG4-related lesion. Her elevated serum IgG4 level and a past history of sicca complex supported the diagnosis of IgG4-RD.

**Conclusions:**

It is critical to recognize the importance of laboratory examinations such as serum IgG4 level if a patient has a past history of rheumatic disease.

## Background

Immunoglobulin G4–related disease (IgG4-RD) is a new clinical entity. Characteristic features of IgG4-RD are elevated serum IgG4 levels, infiltration of IgG4 positive cells, mass-forming lesions with fibrosis and good response to corticosteroids. Variable imaging features of IgG4-RD and the overlap with other differential diagnoses often pose a diagnostic challenge, as they frequently mimic malignant tumors or other inflammatory diseases in the abdomen.

## Case presentation

**Figure 1:**
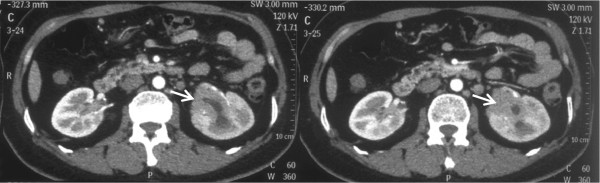
**Abdominal computed tomographic scans.** These scans show a low-density renal pelvic mass (white arrows) and hydronephrosis of the left kidney.

**Figure 2:**
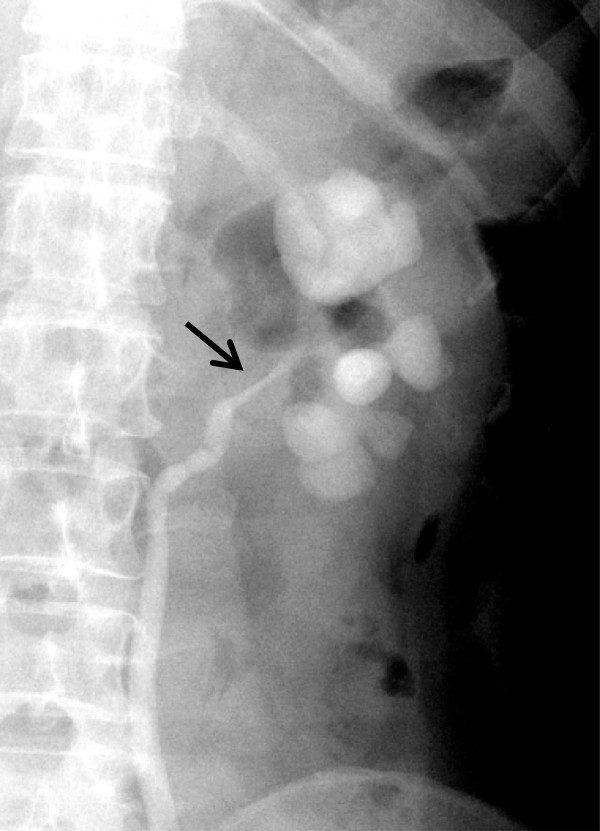
**Retrograde pyelogram.** This scan shows stricture (arrow) of the left ureteropelvic junction and hydronephrosis.

**Figure 3:**
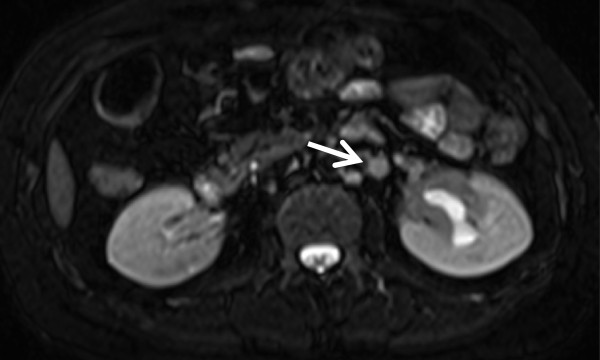
**Abdominal T2-weighted magnetic resonance imaging study.** This scan shows a low-density renal pelvic mass and hydronephrosis of the left kidney. An enlarged retroperitoneal lymph node (arrow) can be seen.

**Figure 4:**
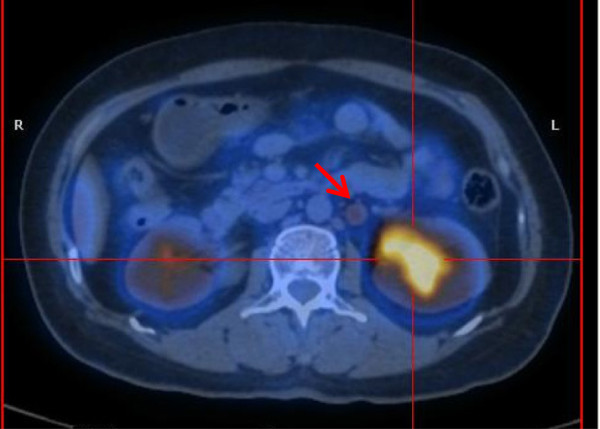
**Positron emission tomography/computed tomography.** This scan shows a hypermetabolic renal pelvic mass and an enlarged retroperitoneal lymph node (arrow).

A 54-year-old woman presented at our hospital with left flank discomfort and palpebral edema of 1-week duration. She underwent abdominal postcontrast computed tomography (CT) in a local hospital, which showed a low-density renal pelvic mass and hydronephrosis of the left kidney and indicated renal pelvic cancer (Figure 1). Her past medical history included sicca complex for 5 years previously. In her laboratory examination, a routine urine test revealed a red blood cell count of 118.4/μl (normal reference range, 0 to 25/μl), a white blood cell count of 127.3/μl (normal reference range, 0 to 25/μl) and an epithelial cell count of 13.4/μl (normal reference range, 2 to 10/μl). No remarkable findings in the complete blood count or urine cytology were observed. A retrograde pyelogram showed a dilated left renal pelvis and stricture of the upper ureter, which had a regular surface and a filling defect (Figure 2). On postcontrast magnetic resonance imaging (MRI) scans, the wall of the ureteropelvic junction was irregularly thickened and showed isointensity on T1-weighted images and hypointensity on T2-weighted images. On both T1- and T2-weighted images, the thickened wall of ureteropelvic junction showed homogeneous enhancement. Furthermore, multiple enlarged retroperitoneal lymph nodes were visualized by MRI (Figure 3). Positron emission tomography/CT findings indicated that the renal pelvic mass was a malignant tumor, because the glucose metabolism was very high (Figure 4). PET/CT also revealed multiple enlarged hypermetabolic lymph nodes in the supraclavicular, retroperitoneal, peritoneal and pelvic regions. All of these findings together led us to consider a possible diagnosis of a renal pelvic malignant tumor with multiple lymph nodes metastasis.

A few days later the patient underwent a left-sided nephroureteral cystectomy and retroperitoneal lymph node dissection, in which part of the bladder was removed. The surgery was performed to establish a definitive diagnosis and for treatment if the mass was malignant.

**Figure 5:**
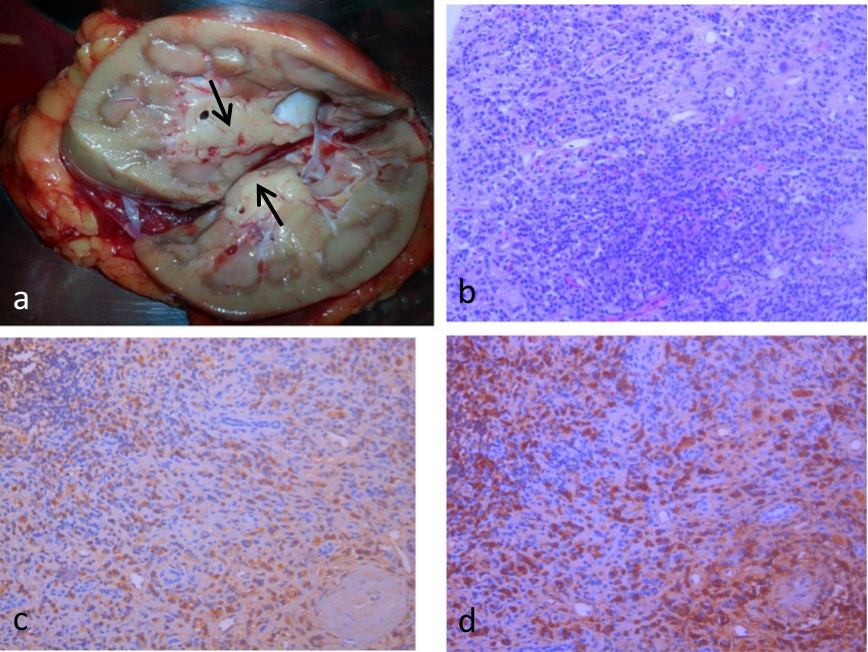
**Postoperative images. (a)** Gross examination of the kidney showed a 5 × 2.5–cm, pale, whitish-tan, ill-defined mass located in the renal pelvis near the renal hilum. **(b)** Histological section of the renal mass shows lymphatic tissue hyperplasia and diffuse infiltration of plasma cells. **(c)** Histological section of the renal mass showed immunoglobulin G (IgG)-positive plasma cells (anti-IgG antibody stain). **(d)** Histological section of the renal mass showed IgG4-positive plasma cells (anti-IgG4 antibody stain). The IgG4/IgG ratio was approximately 40%.

Gross examination of the kidney showed a 5 × 2.5–cm, pale, whitish-tan, ill-defined mass located in the renal pelvis near the renal hilum. Histologic examination of the mass showed lymphatic tissue hyperplasia and diffuse infiltration of plasma cells. The plasma cells were IgG- and IgG4-positive. The IgG4/IgG ratio was approximately 40% (Figure 5). Two retroperitoneal lymph nodes were dissected, which represented as reactive hyperplasia. The pathological findings did not reveal malignancy.

After the diagnosis of IgG4-RD was made, a further laboratory examination was performed. The patient’s serum IgG4 level was 18.6 g/L (normal reference range, 0.03 to 2 g/L), and her high-sensitivity C-reactive protein test result was 26.3 mg/L (normal reference range, 0 to 3 mg/L). The patient underwent steroid therapy in a local hospital for 1 year. At the fourth month of her steroid therapy, her high-sensitivity C-reactive protein level had descended to 8.76 mg/L (normal reference range, 0 to 8 mg/L). Her enlarged lymph nodes in the supraclavicular, retroperitoneal, peritoneal and pelvic regions diminished with the steroid therapy.

## Discussion

IgG4-RD is a relatively new clinical entity proposed by authors from Japan [1]. Characteristic features of IgG4-RD are elevated serum IgG4 level, infiltration of IgG4 positive cells, mass-forming lesions with fibrosis and good response to corticosteroids.

The variable imaging features of IgG4-RD and the overlap with other differential diagnoses often pose a diagnostic challenge, as they frequently mimic malignant tumors or other inflammatory diseases in the abdomen [2]. Renal lesions in IgG4-RD most commonly manifest as multiple wedge-shaped or nodular cortical lesions in one or both kidneys [3]. These lesions are of low intensity in the early phase of postcontrast CT or MRI scans and become less distinct in the late phase of enhancement. The lesions are typically hypointense on T2-weighted MRI scans. Hematuria may often be observed [2, 4].

IgG4-related renal lesions can mimic renal cell carcinoma or transitional cell carcinoma in uncommon cases of a single unilateral mass, such as a renal lesion or focal unilateral renal pelvic wall thickening. Yoshino *et al*. [5] reported a case of IgG4-related retroperitoneal fibrosis mimicking renal pelvic cancer. CT revealed left hydronephrosis and a thick retroperitoneal soft tissue mass around the ureteropelvic junction, raising suspicion of renal pelvic cancer. Their patient refused tumor biopsy. Therefore, he was treated with corticosteroid therapy on the basis of a clinical diagnosis of IgG4-related retroperitoneal fibrosis. Regression of the retroperitoneal mass and a decrease in serum IgG4 levels were observed.

In our present case, all the imaging findings, including CT, MRI and retrograde pyelography, strongly indicated that the mass was a malignant one. On the basis of our experience in the present case, we do not believe PET/CT is a reliable way to differentiate IgG4-RD from renal malignancy. The renal pelvic mass in our patient was observed to be hypermetabolic on a PET scan. Multiple lymph nodes in the supraclavicular, retroperitoneal, peritoneal and pelvic regions were enlarged and hypermetabolic, vividly mimicking lymph nodes metastasis. We may draw a conclusion that a tumor biopsy should be done for patients with renal pelvic masses, even in cases with confirmed IgG4-RD, on the basis of the fact that the standardized incidence ratio for cancers in patients with IgG4-RD has been reported to be approximately 3.5 times higher than the incidence of cancer in the general population [6].

Uehara *et al*. [7] reported a case of a 66-year-old man with IgG4-RD in whom they performed an autopsy. The patient had been diagnosed with Mikulicz’s disease and an IgG4-related pseudoinflammatory tumor in the pelvis, but the autopsy disclosed the coexistence of diffuse, large B cell lymphoma. Yoshino *et al*. [5] also reported a case of IgG4-RD mimicking renal pelvic cancer. CT in their patient raised suspicions of renal pelvic cancer. Their patient’s serum levels of IgG and IgG4 were found to be elevated in laboratory tests. They treated the patient with corticosteroid therapy. Regression of the retroperitoneal mass and improvement of left hydronephrosis were achieved. Our patient responded to corticosteroid therapy, too, with a decline in high-sensitivity C-reactive protein and diminishment of enlarged lymph nodes. Because conservative treatments have remarkable effect in this kind of disease, it is vital to avoid unnecessary surgery. CT-guided biopsy or laparoscopic biopsy of the original tumor or lymph node swelling might help to rule out malignancy.

The past medical history is another important clue in the differentiation of IgG4-RD from renal pelvic malignancy. The salivary gland is one of the target organs in IgG4-RD, and the clinical phenotype is so-called Mikulicz’s disease [8]. In the present case, our patient’s history of sicca complex did not draw much attention, because the imaging findings strongly indicated a malignant tumor.

## Conclusions

In patients such as the one we describe in this report, it is critical to recognize the importance of laboratory examinations such as serum IgG4 level if a patient has a past history of rheumatic disease.

## Consent

Written informed consent was obtained from the patient for the publication of this report and any accompanying images.
